# Using fNIRS to Capture Cerebral Oxygenation in Older Adults Navigating Stairs

**DOI:** 10.1093/geroni/igaa057.2870

**Published:** 2020-12-16

**Authors:** Sarah Fraser, Talia Salzman, Hyejun Kim, Hawazin Badawi, Diana Tobon Vallejo, Yves Lajoie, Lara Pilutti, John Farrell

**Affiliations:** 1 University of Ottawa, Ottawa, Ontario, Canada; 2 Universidad de Medellin, Medellin, Antioquia, Colombia

## Abstract

Navigating stairs is a complex motor activity and while it provides health benefits it can also increase the risk of falls in older adults (OA). The prefrontal cortex (PFC) contributions to stairclimbing (with or without a cognitive task) remain unknown. Using functional near infra-red spectroscopy (fNIRS) and wireless insoles, this study evaluated cerebral oxygenation changes (∆HbO2) in the PFC, gait parameters (speed) and cognitive performance (reaction time(RT)/accuracy) during stair ascent and descent in single (SMup/SMdown) and dual task (DTup/DTdown) conditions. OAs navigated stairs with or without a simple reaction time task. Participants had longer RTs in DTup (p < .001) and DTdown (p <.001) in comparison to standing, with no significant differences in accuracy or walk speed. ∆HbO2 was significantly different (p = .003) between SMdown and DTdown. Findings suggest that despite the simplicity of the cognitive task, dual-tasking on stairs resulted in increased cerebral oxygenation and slowed cognitive responses.

